# Sterol C-22 Desaturase ERG5 Mediates the Sensitivity to Antifungal Azoles in *Neurospora crassa* and *Fusarium verticillioides*

**DOI:** 10.3389/fmicb.2013.00127

**Published:** 2013-05-29

**Authors:** Xianyun Sun, Wenzhao Wang, Kangji Wang, Xinxu Yu, Jie Liu, Fucai Zhou, Baogui Xie, Shaojie Li

**Affiliations:** ^1^State Key Laboratory of Mycology, Institute of Microbiology, Chinese Academy of SciencesBeijing, China; ^2^Mycological Research Center, College of Life Sciences, Fujian Agriculture and Forestry UniversityFuzhou, China; ^3^College of Bioscience and Biotechnology, Yangzhou UniversityYangzhou, China

**Keywords:** resistance to antifungal agents, ergosterol biosynthesis, azole, *Neurospora crassa*, *Fusarium verticillioides*

## Abstract

Antifungal azoles inhibit ergosterol biosynthesis by interfering with lanosterol 14α-demethylase. In this study, seven upregulated and four downregulated ergosterol biosynthesis genes in response to ketoconazole treatment were identified in *Neurospora crassa*. Azole sensitivity test of knockout mutants for six ketoconazole-upregulated genes in ergosterol biosynthesis revealed that deletion of only sterol C-22 desaturase ERG5 altered sensitivity to azoles: the *erg5* mutant was hypersensitive to azoles but had no obvious defects in growth and development. The *erg5* mutant accumulated higher levels of ergosta 5,7-dienol relative to the wild type but its levels of 14α-methylated sterols were similar to the wild type. ERG5 homologs are highly conserved in fungal kingdom. Deletion of *Fusarium verticillioides* *erg5* also increased ketoconazole sensitivity, suggesting that the roles of ERG5 homologs in azole resistance are highly conserved among different fungal species, and inhibition of ERG5 could reduce the usage of azoles and thus provide a new target for drug design.

## Introduction

Antifungal azoles are the most commonly used drugs for controlling fungal infections. Azoles inhibit ergosterol biosynthesis by disrupting essential P450 superfamily protein lanosterol 14α-demethylase CYP51 (syn. ERG11) (Bossche et al., 1984; Kelly et al., 1993). In addition, the inhibition of lanosterol 14α-demethylase also results in the accumulation of the toxic 14-α methylated sterols, like 14α-methyl-ergosta-8,24(28)-dien-3β,6α-diol (Kelly et al., 1995). Incorporation of such compounds into fungal membranes reduces their rigidity (Abe et al., 2009).

Biological processes of sterol biosynthesis are highly conserved in fungal kingdom (Ferreira et al., 2005). In response to azole treatments, significant transcriptional increases in several genes involved in ergosterol biosynthesis, such as *ERG2* (C-8 sterol isomerase), *ERG3* (C-5 sterol desaturase), *ERG5* (C-22 sterol desaturase), *ERG6* (C-24 sterol methyltransferase), *ERG25* (C-4 methyl sterol oxidase), and *ERG11*, are consistently observed in many fungal species such as *Saccharomyces cerevisiae* (Bammert and Fostel, 2000; Agarwal et al., 2003), *Candida albicans* (De Backer et al., 2001; Liu et al., 2005), *Aspergillus fumigatus* (da Silva Ferreira et al., 2006), *Trichophyton rubrum* (Yu et al., 2007), *Cryptococcus neoformans* (Florio et al., 2011), and *Fusarium graminearum* (Liu et al., 2010; Becher et al., 2011). Many reports have shown that increased expression of *ERG11* could reduce azole susceptibility (Hamamoto et al., 2000; Schnabel and Jones, 2001; Du et al., 2004; Ma et al., 2006). In addition to *ERG11*, *ERG3*, and *ERG5* have also been shown to be related to drug susceptibility. Many *ERG3* mutants of *S. cerevisiae* or *C. albicans* with defects in C5-6 desaturase showed increased resistance to azoles (Watson et al., 1989; Martel et al., 2010a; Morio et al., 2012; Vale-Silva et al., 2012). A clinical isolate of *C. albicans* with mutations in both *ERG11* and *ERG5* is cross resistance to azoles and amphotericin B (Martel et al., 2010b). However, due to lack of systemic analysis, except *ERG11*, contributions of most of these azole-responsive genes to azole resistance were not fully understood.

Since the non-pathogenic *Neurospora crassa* has less redundant genes in ergosterol biosynthesis relative to other filamentous pathogenic fungi and has knockout mutants available for most of ergosterol synthesis genes, it can be an excellent model for systemic analysis of the contributions of these azole-responsive genes to azole resistance. In this study, we demonstrate that ERG5 mediates the sensitivity to antifungal azoles in both *N. crassa* and *Fusarium verticillioides*.

## Materials and Methods

### Strains and culture conditions

*Neurospora crassa* strains used in this study are listed in Table 1. All of them were obtained from the Fungal Genetics Stock Center^1^ (University of Kansas Medical Center). Vogel’s minimum medium (Vogel, 1956), supplemented with 2% (w/v) sucrose for slants or 2% glucose for plates and the liquid medium, was used for culturing strains. All cultures were grown at 28°C in the light. Antifungal compounds were added as needed.

**Table 1:** ***Neurospora* strains used in this study**.

Strain	Genotype
FGSC#4200	Wild type, a
FGSC#18507	*erg2* (NCU04156), a, hetero
FGSC#13802	*erg5* (NCU05278), a
FGSC#13803	*erg5* (NCU05278), A
FGSC#12752	*erg6* (NCU03006), a
FGSC#12753	*erg6* (NCU03006), A
FGSC#18506	*erg24* (NCU08762), a, hetero
FGSC#17674	*erg25* (NCU06402), a

### Drug susceptibility test

Ketoconazole, fluconazole, and itraconazole were dissolved in dimethyl sulfoxide (DMSO) and then aseptically added to the autoclaved medium before making agar plates. The final concentrations of ketoconazole, fluconazole, and itraconazole in the agar plates were 2, 25, and 10 μg/ml, respectively. The final DMSO concentration was below 0.25% (v/v). Two microliters of conidial suspension were inoculated on plates (Φ = 9 cm) with or without antifungal drugs and incubated at 28°C for 66 h.

### MIC determination of ketoconazole, itraconazole, and fluconazole

The MICs of three azoles for each strain were determined in 96-well microtiter plate according to National Committee for Clinical Laboratory Standards (CLSI, 2008). Briefly, 100 μl of 2× azole solution and 100 μl of conidial suspension were added into each well. The final conidial concentration was approximately 1 × 10^6^ conidia/ml. The plates were incubated at 28°C for 24 h. The MIC was determined as the lowest drug concentration without growth.

### RNA extraction and digital gene expression profiling analysis

The transcriptional responses of ergosterol biosynthesis genes to ketoconazole treatment in the wild-type strain were detected by digital gene expression (DGE) profiling (Nielsen et al., 2006). Briefly, conidia of the wild-type strain was inoculated into 20-ml liquid medium in a plate (Φ = 9 cm) and incubated for 24 h at 28°C in darkness to form mycelial mat on the surface of the liquid medium. Mycelial mat then was cut into small pieces (Φ = 10 mm) and transferred to the liquid medium (2 pieces/100 ml). Cultures were incubated at 28°C with shaking at 180 rpm for 12 h. Ketoconazole was then added into the medium to reach a final concentration of 2.5 μg/ml. After 24 h of incubation, mycelia were harvested and total RNA was extracted and subjected to DGE analysis as described by Sun et al. (2011).

### Sterol extraction and analysis

Sterols of dried mycelium (0.3 g) was extracted into 1.5-ml chloroform under ultrasonication for 12 h. The extracts were dried and subsequently dissolved into 150 μl methanol under ultrasonication for 1 h. After filtration with a Millipore filter, the extracts were subjected to HPLC-MS analysis following the method reported by Cañabate-Díaz et al. (2007) with some modifications.

Liquid chromatography separation was performed on an Agilent Zorbax Extend-C18 1.8 μm 2.1 mm × 50 mm column using an Agilent 1200 Series system (Agilent, USA). Total flow rate was 0.4 ml min^−1^; mobile phase A consisted of water with 0.01% acetic acid and mobile phase B consisted of acetonitrile. Total elution program was 27 min. Gradient began with 70% mobile phase B, changed to 100% B over 2 min, maintained a constant level from 2 to 18 min at 100% B and then decreased to 70% B over 1 min prior to re-stabilization over 8 min before the next injection. The column temperature was maintained at 40°C. The injection volume was 10 μl.

Mass spectra were acquired with an Agilent Accurate-Mass-Q-TOF MS 6520 system. Analytes were detected in the positive ionization mode with an APCI probe. For Q-TOF/MS conditions, fragmentor and capillary voltages were kept at 130 and 3500 V, respectively. Nitrogen was supplied as the nebulizing and drying gas. Temperature of the drying gas and vaporizer were both set at 350°C. The corona was set to 4 μA. The flow rate of the drying gas and the pressure of the nebulizer were 3 l min^−1^ and 50 psi, respectively. Full-scan spectra were acquired over a scan range of *m/z* 80–1000 at 1.03 spectra s^−1^. The derived sterols were identified with reference molecular weight and fragmentation spectra for known standards.

### Phylogenetic analysis

Protein sequences were aligned with the Clustal X 2.1 software (Larkin et al., 2007). Then, the neighbor-joining (NJ) tree was constructed using MEGA 5 software (Tamura et al., 2011). To assess the confidence of phylogenetic relationships, the bootstrap tests were conducted with 1000 resamplings.

### Knockout of *erg*5 homolog gene in *Fusarium verticillioides*

In order to knockout *erg5* homologous genes (*erg5A*: FVEG_07284; *erg5B*: FVEG_08786) in *F. verticillioides*, the upstream and downstream flanking sequences of the FVEG_07284 and FVEG_08786 were amplified. The primer pairs were shown in Table 2.

**Table 2:** **Primer pairs used for knockout of *erg5* homolog gene in *F. verticillioides***.

Primers	Sequence (5′ → 3′)	Product size (bp)	Amplified region
Fv07284(p)F-*Xho*I	CCGCTCGAGCACCCGATGAACTCGCCAATA	1859	FVEG_07284 5′ flanking region
Fv07284(p)R-*Eco*RI	CGGAATTCATCATACGCAACGCAAAGAGC	
Fv07284(3)F-*Bam*HI	CGGGATCCATGATGGGAAAGCGAGTTGA	1717	FVEG_07284 3′ flanking region
Fv07284(3)R-*Xba*I	GCTCTAGAGCTGACAGCGACCAGTAGGA	
Fv08786(p)F-*Kpn*I	GCGGTACCCGAGGATGATTGCTTGGTGAG	1455	FVEG_08786 5′ flanking region
Fv08786(p)R-*Eco*RI	CGGAATTCCATGCTGGGTCTAGTTGAGGG	
Fv08786(3)F-*Xba*I	GCTCTAGAGGGGCAAGGTGTTTGTGAATA	1797	FVEG_08786 3′ flanking region
Fv08786(3)R-*Xba*I	GCTCTAGAAATGCCACTGAGTTCGGATG	

The resulting PCR products were cloned into the plasmid pCX62 (Zhao et al., 2004) and results in knockout construct pCX62-ΔFv07284 and pCX62-ΔFv08786, in which the PCR products were ligated with the hygromycin phosphotransferase (*hph*) gene. Then, the deletion cassette was transformed into *F. verticillioides* 7600 and resulted in deletion mutants. Fungal transformation followed the protocol reported by Miller et al. (1985), with minor modification as described by Li et al. (2006).

## Results

### Transcriptional responses to ketoconazole stress by genes involved in ergosterol biosynthesis

Genome-wide transcriptional responses to ketoconazole treatment from two independent experiments were analyzed by DGE method. For genes involved in ergosterol biosynthesis, seven were consistently upregulated while four consistently downregulated upon ketoconazole treatment in two independent experiments (Table 3). The most dramatically upregulated genes were *erg11* (NCU2624), *erg6* (NCU03006), *erg2* (NCU04156), and *erg5* (NCU05278), which were consistently increased upon ketoconazole treatment by at least three folds in two independent experiments. The consistently downregulated genes include *erg7* (NCU01119), *erg8* (NCU08671), *erg13* (NCU03922), and *erg20* (NCU11381). However, the transcriptional changes in these downregulated genes were less dramatic than those in *erg11*, *erg6*, *erg2*, and *erg5*. None of them had a transcriptional change higher than three folds.

**Table 3:** **Transcriptional response to ketoconazole stress by genes involved in ergosterol biosynthesis in *N. crassa* wild type**.

Locus	Gene	Function	TPM-wt1	TPM-wt(k)1	Fold [wt(k)1/wt1]	TPM-wt2	TPM-wt(k)2	Fold [wt(k)2/wt2]
NCU08280	*erg1*	Squalene epoxidase	78.1	34.6	0.44	16.1	27.4	1.70
NCU04156	*erg2*	Sterol biosynthesis	107.1	327.7	3.06	26.8	296.4	11.07
NCU06207	*erg3*	C-5 sterol desaturase	1000.3	767.0	0.77	639.5	999.0	1.56
NCU01333	*erg4*	C-24 reductase	1.4	0.6	0.42	1.4	2.0	1.36
NCU05278	*erg5*	C-22 sterol desaturase	67.4	308.9	4.58	83.5	174.6	2.09
NCU03006	*erg6*	C-24 sterol methyltransferase	23.8	791.5	33.20	19.3	174.6	9.06
NCU01119	*erg7*	Oxidosqualene:lanosterol cyclase/lanosterol synthase	34.0	11.3	0.33	23.0	4.5	0.19
NCU08671	*erg8*	Phosphomevalonate kinase	24.7	8.7	0.35	26.5	10.6	0.40
NCU06054	*erg9*	Squalene synthetase	21.1	14.7	0.70	10.1	14.6	1.44
NCU02571	*erg10*	Acetoacetyl-CoA thiolase	181.6	178.8	0.98	323.8	103.8	0.32
NCU02624	*erg11*	Cytochrome P450 lanosterol 14α-Demethylase	110.1	812.0	7.37	69.1	676.7	9.80
NCU03633	*erg12*	Mevalonate kinase	70.1	90.6	1.29	21.6	77.5	3.59
NCU03922	*erg13*	Hydroxymethylglutaryl-coenzyme A synthase	117.0	47.3	0.40	227.4	35.0	0.15
NCU11381	*erg19*	Mevalonate pyrophosphate decarboxylase	38.6	21.1	0.55	49.5	18.2	0.37
NCU01175	*erg20*	Polyprenyl synthetase	188.5	81.1	0.43	116.0	50.9	0.44
NCU08762	*erg24*	Sterol C-14 reductase	7.1	11.8	1.66	9.2	34.7	3.77
NCU06402	*erg25*	C-4 methyl sterol oxidase	2512.8	5111.7	2.03	2894.3	5348.9	1.85
NCU02693	*erg26*	C-3 sterol dehydrogenase	57.0	63.2	1.11	81.5	48.4	0.59
NCU05991	*erg27*	3-Keto sterol reductase	3.0	4.6	1.53	0.6	1.7	2.90
NCU04461	*erg28*	Endoplasmic reticulum membrane protein	67.7	28.9	0.43	30.2	33.3	1.10
NCU04144	*are2*	Acyl-CoA:sterol acyltransferase	1.4	1.4	1.05	0.6	0.1	0.17
NCU00712	*hmg1/hmg2*	Hydroxymethylglutaryl-coenzyme A reductase	14.8	8.1	0.55	9.8	10.1	1.03
NCU07719	*idi1*	Isopentenyl diphosphate isomerase	59.2	15.6	0.26	39.4	37.5	0.95
NCU08139	*mvd1*	Diphosphomevalonate decarboxylase	0	0	–	0	0	–

*wt1 and wt(k)1 represent the first batch samples, wt2 and wt(k)2 represent the second batch samples; wt: wild type; wt(k): wild type treated with ketoconazole*.

### Deletion of *erg*5 increases azole susceptibility in *N. crassa*

**Figure 1:**
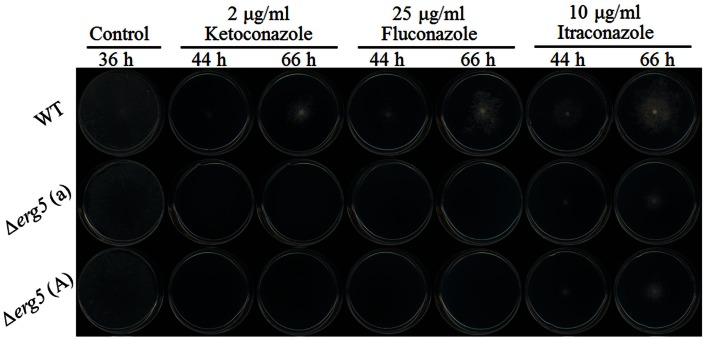
**Drug susceptibility analysis of the *erg5* knockout mutant of *N. crassa***. Two microliters of conidial suspension (1 × 104) conidia/ml were inoculated on the center of plates (Φ = 9 cm) with or without antifungal drugs, then incubated at 28°C for 66 h. Each test had three replicates and the experiment was independently repeated twice.

Six ketoconazole-responsive genes involved in ergosterol synthesis have the knockout mutants available in Fungal Genetics Stock Center. All these genes are ketoconazole-upregulated genes, including *erg2*, *erg3*, *erg5*, *erg6*, *erg24*, and *erg25*. On the solid medium without antifungal drugs, all deletion mutants had the growth rates similar to the wild-type strain and no obvious defect was observed in their asexual and sexual reproduction (the data not shown)^2^. Drug sensitivity test showed that the deletion of only *erg5* altered the sensitivity to azoles. On agar plates, all three tested azoles, including ketoconazole, itraconazole, and fluconazole, had greater inhibition to the *erg5* mutants than the wild-type strain (Figure 1). MIC analysis in the liquid medium showed that MIC values of the *ERG5* mutants for ketoconazole, itraconazole, and fluconazole were only 60, 50, and 40% of that of the wild-type strain, respectively (Table 4).

**Table 4:** **MIC of the *erg5* mutant and wild type for azoles**.

	MIC for ketoconazole (μg/ml)	MIC for fluconazole (μg/ml)	MIC for itraconazole (μg/ml)
*WT*	2.5 ± 0	31.25 ± 0	5.0 ± 0
*Δerg5 (a)*	1.5 ± 0	12.5 ± 0	2.5 ± 0
*Δerg5 (A)*	1.5 ± 0	12.5 ± 0	2.5 ± 0

### Deletion of *erg*5 does not cause accumulation of 14α-methylated sterols during ketoconazole stress in *N. crassa*

**Figure 2:**
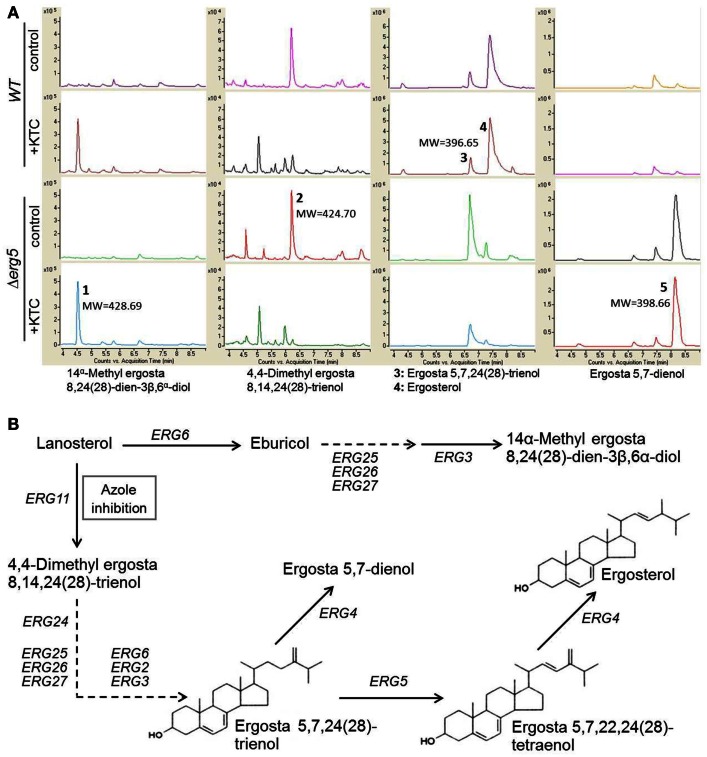
**HPLC-MS chromatogram of sterol extracts (A) and schematic representation of the ergosterol biosynthetic pathway (B) in *N. crassa***.

ERG5 catalyzes the biosynthesis of ergosta 5,7,22,24(28)-trienol, a direct precursor for ergosterol biosynthesis. ERG5 mutations caused accumulation of ergosta 5,7,24(28)-trienol and ergosta 5,7-dienol in *S. cerevisiae* *ERG5* deletion mutant (Barton et al., 1974; Skaggs et al., 1996). Thus, it is possible that the *erg5* mutant might accumulate more toxic 14α-methylated sterols than the wild-type strain. To test this possibility, a comparative analysis of sterol profiles between the *erg5* mutant and the wild type was performed by HPLC-MS. Our results showed that, in the normal liquid medium without ketoconazole, the *erg5* mutant produced more ergosta 5,7-dienol than the wild-type strain. As expected, ergosterol in the *erg5* mutant was almost undetectable (Figure 2). When treated with ketoconazole, 4,4-dimethyl-ergosta 8,14,24(28)-trienol, the direct product catalyzed by ERG11 (lanosterol 14α-methylase), was reduced in the wild-type strain and the *erg5* mutant at the similar level. Ketoconazole treatment greatly increased levels of 14α-methylated sterols, such as eburicol and 14α-methyl-ergosta-8,24(28)-dien-3β, 6α-diol, in both the wild-type strain and the *erg5* mutant (Figure 2). However, the levels of these compounds were similar between the wild-type strain and the *erg5* mutant, suggesting that the azole-hypersensitive phenotype in the *erg5* mutant is not due to over-accumulation of 14α-methylated sterols.

### ERG5 homologs are highly conserved in fungal kingdom

**Figure 3:**
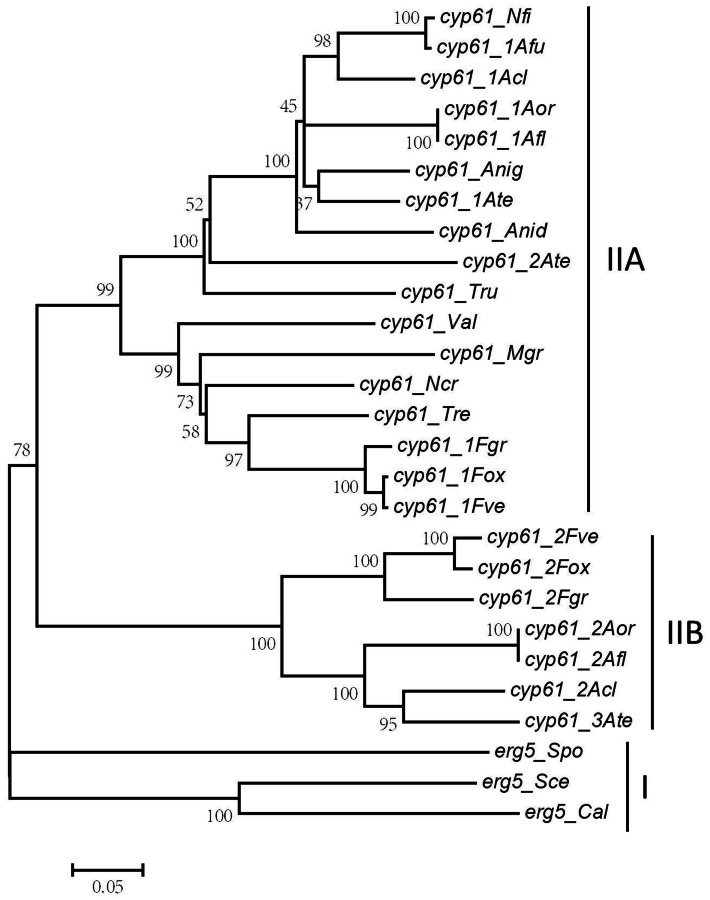
**Phylogenic analysis of fungal ERG5 homologs**. Predicted REG5 proteins from 19 fungal species were aligned and a condensed neighbor-joining (NJ) tree was constructed with cut-off bootstrap values of 50% obtained from 1000 replicates using MEGA 5 software (Tamura et al., 2011). (*Cal*, *C. albicans*; *Sce*, *S. cerevisiae*; *Spo*, *S. pombe*; *Ncr*, *N. crassa*; *Afu*, *A. fumigatus*; *Afl*, *A. flavus*; *Anid*, *A. nidulans*; *Anig*, *A. niger*; *Acl*, *A. clavatus*; *Ate*, *A. terreus*; *Aor*, *A. oryzae*; *Nfi*, *N. fischeri*; *Fve*, *F. verticillioides*; *Fox*, *F. oxysporum*; *Fgr*, *F. graminearum*; *Tru*, *T. rubrum*; *Val*, *V. albo-atrum*; *Tre*, *T. reesei*; *Mag*, *M. grisea*).

To gain an insight into the sequence conservation and phylogenic relationship of ERG5 homologs in fungi, Blast and phylogenetic analysis were conducted. Blast analysis revealed that ERG5 homologs are high conserved in amino acid sequences. Phylogenetic analysis with amino acid sequences of 27 ERG5 homologs (*E* value = 0; Alignment coverage >90%; sequence similarity >76%) from 3 yeast fungal species (*C. albicans*, *S. cerevisiae*, *Schizosaccharomyces pombe*) and 16 filamentous fungal species showed that all ERG5 homologs were clustered into 2 big clades (Figure 3). The ERG5 homologs from yeast fungal species were distributed in the first clade (clade I), which represents the most ancestral group, while those from filamentous fungal species were located in the second clade (clade II).

As shown in Figure 3, ERG5 in filamentous fungi can be further categorized into two classes based on their phylogenic relationship, namely Type IIA and Type IIB, respectively. *N. crassa*, *N. fischeri*, *A. niger*, *A. nidulans*, *T. reesei*, *M. grisea*, and *V. albo-atrum* have only one *erg5* gene, all in Type IIA. *A. fumigatus*, *A. oryzae*, and *A. clavatus* and all examined *Fusarium* species have two *erg5* genes, one in Type IIA and the other in Type IIB. *A. terreus* is the only species that has three *erg5* genes, two in Type IIA and one in Type IIB. Similar to ERG5, multiple ERG3 and ERG11 homologs were also found in *Aspergillus* and *Fusarium* species (Ferreira et al., 2005; Liu et al., 2010; Becher et al., 2011).

### Deletion of *erg*5 increases azole susceptibility in *F. verticillioides*

**Figure 4:**
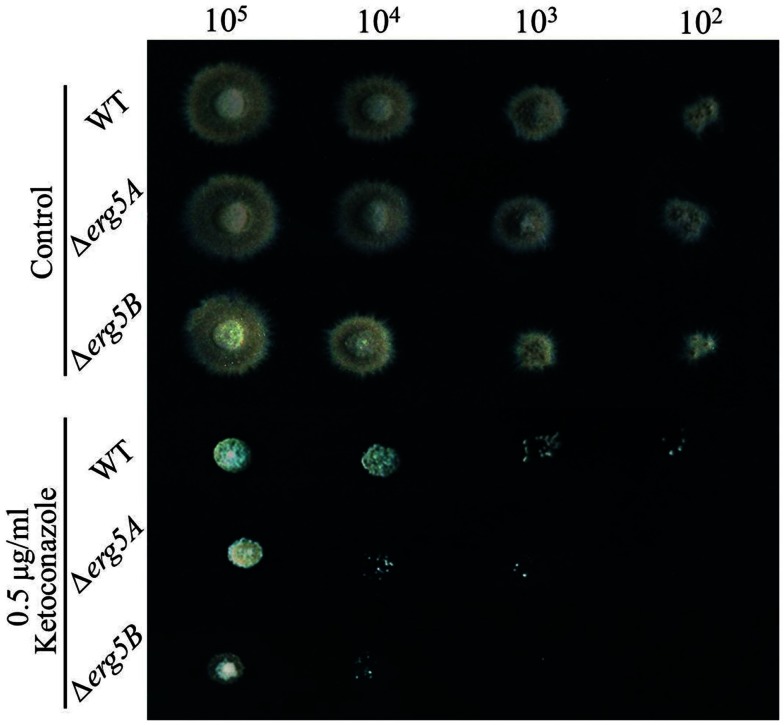
**Drug susceptibility analysis of the *erg5A* (FVEG_07284) and *erg5B* (FVEG_08786) knockout mutants of *F. verticillioides***. Two microliters of conidial suspensions with different concentration (1 × 105, 1 × 104, 1 × 103, or 1 × 102 conidia/ml, respectively) were inoculated on the plates (Φ = 9 cm) with or without antifungal drugs, and incubated at 28°C for 72 h. Each test had three replicates and the experiment was independently repeated twice.

To see if ERG5 in other filamentous fungi also mediate azole sensitivity, gene knockout mutants for Type IIA *erg5* gene *erg5A* (FVEG_07284) and for Type IIB *erg5* gene *erg5B* (FVEG_08786), in the pathogenic fungus *F. verticillioides* were generated. As shown in Figure 4, on the medium without drug, the growth rates of the *erg5* deletion mutants were similar to that of the wild-type strain and no defect in conidiation was observed in the mutants. When inoculated on the medium with 0.5 μg/ml ketoconazole, both *erg5A* and *erg5B* mutant displayed greater growth inhibition than the wild-type strain, indicating the ERG5 homologs have similar roles in azole resistance among different fungal species.

## Discussion

Although transcriptional responses by genes of sterol biosynthesis to azole stress have been observed in many fungal species (Bammert and Fostel, 2000; De Backer et al., 2001; Agarwal et al., 2003; Liu et al., 2005, 2010; da Silva Ferreira et al., 2006; Yu et al., 2007; Becher et al., 2011; Florio et al., 2011), it is still mysterious about the biological significance of transcriptional activation for most of these genes. Taking advantage of the availability of knockout mutants of *N. crassa*, we analyzed the contributions of six ketoconazole-responsive genes in ergosterol biosynthesis, including *erg2*, *erg3*, *erg5*, *erg6*, *erg24*, and *erg25*, to azole resistance, and demonstrated that deletion of *erg5*, but not other ergosterol genes, could make *N. crassa* more susceptible to antifungal azoles. Since the deletion of *erg5* should result in an effect opposite to its overexpression, transcriptional increase of *erg5* upon azole stress might positively contribute to the azole resistance. As *erg5* deletion consistently resulted in azole hypersensitivity in both *N. crassa* and *F. verticillioides*, strategies that either silencing the expression of *erg5* or disrupting C-22 sterol desaturase (ERG5) activity can make fungal pathogens more susceptible to antifungal azoles. ERG5, thus, can be a promising target for antifungal drug design or fungal disease management in crops. Although no alteration in azole sensitivity was observed in the mutants for the rest of ergosterol biosynthesis genes, their transcriptional increases during azole treatment suggest that these genes are involved in the adaptation to azole stress. Their contributions to azole resistance might be able to be detected by combined mutation of multiple genes. Nevertheless, *erg5* is the most important contributor to azole resistance among these azole-responsive genes.

Membrane sterols regulate membrane fluidity, permeability, the activity of membrane-bound enzymes and growth rate (Skaggs et al., 1996). In *S. cerevisiae*, genes involved in early steps of ergosterol biosynthesis, such as *ERG9* (squalene synthase), *ERG1* (squalene epoxidase), *ERG7* (lanosterol synthase), *ERG11* (lanosterol 14α demethylase), and *ERG24* (C-14 reductase), are essential, while genes participating the late steps of ergosterol biosynthesis, such as *ERG2*, *ERG3*, *ERG6*, *ERG5*, *ERG25*, and *ERG4*, are not essential (Fryberg et al., 1973; Lees et al., 1995). In *N. crassa*, viable homokaryotic mutants for only these non-essential genes were generated. Thus, the end-product ergosterol is not necessary for fungal survival. When ergosterol is absent, other sterol intermediates might function as substitutes of ergosterol. Similar to previous observation in *S. cerevisiae* (Kelly et al., 1995), our data showed that disruption of lanosterol 14α-demethylase by ketoconazole reduced 4,4-dimethyl-ergosta 8,14,24(28)-trienol biosynthesis and accumulated 14α methylated sterols such as 14α-methyl-ergosta-8,24(28)-dien-3,6-diol. ERG5 catalyzes the chemical reaction downstream to 4,4-dimethyl-ergosta 8,14,24(28)-trienol biosynthesis. As shown in Figure 2, deletion of *erg5* is not likely to reduce the biosynthesis of 4,4-dimethyl-ergosta 8,14,24(28)-trienol under ketoconazole stress. Therefore, the azole-hypersensitive phenotype in *erg5* mutants is not related to the biosynthesis of 4,4-dimethyl-ergosta 8,14,24(28)-trienol. In addition, deletion of *erg5* did not lead an increase in accumulation of 14α-methylated sterols upon ketoconazole stress. Thus, over-accumulation of 14α-methylated sterols is also not a possible cause to the azole-hypersensitive phenotype in *erg5* mutants. An *in vitro* experiment has shown that ketoconazole can bind to ERG5 (CYP61) of *S. cerevisiae* with an affinity similar to that of ERG11 (Kelly et al., 1997). Binding of azole to ERG5 might reduce the concentration of azoles attacking ERG11, providing a protective buffer to ERG11. If it is true, removal of *erg5* will increase azole sensitivity. In addition, when ergosterol is absent, localization of some membrane proteins, such as azole pumps, might be affected and make cells accumulate more azoles. The real mechanism requires further investigation.

In summary, this study reported azole sensitivities of knockout mutants for 6 azole-responsive genes involved in ergosterol biosynthesis and demonstrated that deletion of *erg5* increase azole sensitivity in both *N. crassa* and *F. verticillioides*. Our findings provide a new insight into in the mechanism of azole resistance.

## Conflict of Interest Statement

The authors declare that the research was conducted in the absence of any commercial or financial relationships that could be construed as a potential conflict of interest.

## Footnotes

^1^www.fgsc.net

^2^http://www.broadinstitute.org/annotation/genome/neurospora/Phenotypes.html
